# Role of symbiotic microbiota dysbiosis in the progression of chronic kidney disease accompanied with vascular calcification

**DOI:** 10.3389/fphar.2023.1306125

**Published:** 2024-01-05

**Authors:** Mengying Sun, Yilong Fang, Jianhua Zheng, Guojuan Shi, Junli Guo, Xinzhou Zhang, Rui Zhang

**Affiliations:** ^1^ Department of Nephrology, Zhuhai People’s Hospital Affiliated with Jinan University, Jinan University, Zhuhai, Guangdong, China; ^2^ School of Pharmaceutical Sciences, Affiliated Foshan Maternity and Child Healthcare Hospital, Southern Medical University, Foshan, China; ^3^ Department of Traditional Chinese Medicine, Huizhou First Hospital, Huizhou, Guangdong, China; ^4^ Department of Nephrology, Shenzhen People’s Hospital, The Second Clinical Medical College of Jinan University, Shenzhen, Guangdong, China

**Keywords:** chronic kidney disease, vascular calcification, gut microbiota, blood microbiota, *Acinetobacter*

## Abstract

**Background:** Chronic kidney disease (CKD) is now globally recognized as a critical public health concern. Vascular calcification (VC) represents a significant risk factor for cardiovascular events in individuals with CKD. It is the accessible and precise diagnostic biomarkers for monitoring the progression of CKD and the concurrent VC are urgently needed.

**Methods:** The adenine diet-induced CKD rat model was utilized to investigate chronic kidney injury, calcification in the kidney and thoracic aorta, and dysregulation of biochemical indices. Enzyme-linked immune sandwich assays were employed to analyze changes in calcification-related proteins. 16S rRNA sequencing was performed to delineate the microbiota characteristics in the gut and blood of CKD-afflicted rats. Additionally, transcriptome sequencing of kidney tissue was conducted to explore the relationship between CKD-associated microbiota features and alterations in kidney function.

**Results:** The adenine diet-induced CKD inhibited body weight gain, and led to kidney injury, and pronounced calcification in kidney and thoracic aorta. The microbiota both in the gut and blood of these affected rats exhibited significantly lower alpha diversity and distinctive beta diversity than those in their healthy counterparts. CKD resulted in dysregulation of several biochemical indices (including elevated levels of creatinine, low-density lipoprotein-cholesterol, sodium, phosphorous, total cholesterol, and urea and decreased levels of albumin, calcium, lactate dehydrogenase, and total bilirubin). Moreover, it upregulated calcification-related factors (bone sialoprotein [BSP], Klotho, fibroblast growth factor [FGF]-23, and sclerostin [SOST]) and lipopolysaccharide (LPS). Notably, the increased *Acinetobacter* in the blood was positively associated with calcifications in the kidney and thoracic aorta, in addition to the positive correlation with gut microbiota. The enrichment of *Acinetobacter* was concurrent with increases in calcification factors (BSP, FGF-23, and SOST), LPS, and phosphorous. Furthermore, transcriptome sequencing revealed that the enrichment of *Acinetobacter* was positively correlated with the majority of upregulated genes and negatively correlated with downregulated genes involved in the mineral absorption pathway.

**Conclusion:** Our findings, for the first time, underscore that dysbiosis of symbiotic microbiota, both in the gut and blood, is involved in the progression of CKD. Particularly, the enrichment of *Acinetobacter* in blood emerges as a potential risk factor for CKD and its accompanying VC.

## 1 Background

Kidney diseases have now been recognized as a global public health priority (Cockwell and Fisher, 2020). Currently, approximately 861 million individuals worldwide suffer from kidney diseases, with the majority presenting with chronic kidney disease (CKD) (Jager et al., 2019). The incidence of CKD in various regions, notably Oceania, sub-Saharan Africa, and Latin America, is significantly higher than expected, irrespective of the extent of development. Furthermore, it imposes a greater health burden on low and middle-income countries than that in affluent ones (Bikbov et al., 2020). CKD is classified into five stages, with renal failure (G5 stage) being the most severe (Zoccali et al., 2023). Cardiovascular events are the leading cause of death in patients with stage G5 CKD undergoing routine dialysis. The heightened cardiovascular risk is largely attributed to vascular calcification (VC) (Zoccali et al., 2023), leading to various fatal complications such as atherosclerosis, heart valve calcification, dyslipidemia regulation, and vasculitis injury. In CKD patients, the primary classical calcification risk factors are hypertension, diabetes, obesity, hyperphosphatemia, hypercalcemia, and dyslipidemia (Li et al., 2020; Qirjazi et al., 2021). Despite the frequent use of radiography and computed tomography scans in surveilling CKD-related cardiovascular calcification, early diagnosis and prevention have not been well implemented due to the absence of suitable biomarkers. Therefore, it is imperative to develop diagnostic biomarkers for the progression of CKD and concomitant VC that are both accessible and precise.

Accumulating evidence reveals that symbiotic microbiota residing in various parts of the body are closely linked to chronic metabolic diseases. The gut microbiota, the largest microecosystem within the host, plays pivotal roles in the development of metabolic disorders, including diabetes (Howard et al., 2022), hypertension (O'Donnell et al., 2023), and hyperlipidemia (Jia et al., 2021), among others. Ren et al. (2020) observed a notable distinction in the microbial community of CKD patients compared to healthy controls. They identified a panel of five microbial markers that could potentially serve as non-invasive diagnostic tools for CKD across various regions of China. Trimethylamine N-oxide, an intriguing metabolite of gut microbiota, has been found to be positively associated with the risk of all-cause mortality in both non-dialysis and non-black dialysis CKD patients (Li et al., 2023). Consequently, interventions targeting gut microbiota dysbiosis may lead to the regulation of gut microbiota, potentially contributing to favorable clinical outcomes in patients with CKD (Sumida et al., 2023).

In addition to the gut microbiota, microbiota in other parts of the body, including the oral cavity, skin, and vagina, also demonstrate versatile interactions with the host’s physiological processes. It has been revealed that alterations in the oral environment can exacerbate the dysbiosis of the symbiotic oral microbiome in CKD patients undergoing peritoneal dialysis (Costa et al., 2021; Baker et al., 2023). Contrary to previous beliefs of sterility, Castillo et al. (2019) demonstrated the presence of a bloodstream microbiome. The existence of commensal gut bacteria in the bloodstream might be attributed to the dysfunctional integrity of subepithelial lamina propria macrophages in colorectal cancer patients (Liu et al., 2018; Rubio and Schmidt, 2018). Notably, Yang et al. (2021) discovered a more diverse blood microbiota among advanced colon cancer patients showing a high response to dendritic cell/cytokine-induced killer cell (DC-CIK) therapy in combination with chemotherapy. Several studies have found dysbiosis of blood microbiota in the progression in CKD. A pilot study by Shah et al. found lower *α* diversity and significant taxonomic variations in the blood microbiome in patients with CKD (Shah et al., 2019). Merino-Ribas et al. (2022) found that gut the *Eubacterium eligens* group (genus) in gut, *Devosia* genus in blood, and circulating sCD14 would be considered as biomarkers for VC, CVD, and mortality risk in CKD. This suggests that the blood microbiome could serve as a potential marker for predicting clinical responses to DC-CIK combined with chemotherapy.

While numerous studies have reported the correlation between gut microbiota dysbiosis and CKD, there has been limited research exploring the role of the blood microbiome in the progression of CKD, as well as its association with accompanying VC. In this present study, we conducted an analysis of the characteristics of gut microbiota and blood microbiota in CKD accompanied by VC to investigate whether there exists an association between symbiotic microbiota dysbiosis and this chronic nephropathy.

## 2 Methods

### 2.1 Animals

All animal experiments were conducted in accordance with the guidelines outlined by the United States National Institutes of Health (NIH Publication, revised 2011) and were approved by the Experimental Animal Ethics Committee of Guangdong Second Traditional Chinese Medicine Hospital (application number 048747). Male Sprague-Dawley (SD) rats (6- to 7-week old), were procured from the Guangdong Medical Laboratory Animal Center in Guangzhou, China. They were housed and raised under specific pathogen-free (SPF) conditions with controlled temperature (23°C ± 2 °C), humidity (50% ± 5%), and a 12- h light/dark cycle and were provided with free access to food and water. All rats were allowed to acclimate for 1 week before the commencement of the experiment.

### 2.2 Animal model of CKD

The adenine diet-induced CKD rat model was established following a 6-week protocol as previously described (Zhao et al., 2019). SD rats were randomly assigned to either the Control group or the Model group, with five rats in each group. Rats in the Model group were fed a standard chow diet supplemented with 0.75% adenine for 6 weeks, while those in the Control group were fed a standard chow diet. Body weights of all rats were recorded weekly. After the 6-week program, feces were collected before blood sample collection from the abdominal aorta. Subsequently, kidneys and thoracic aortas were harvested.

### 2.3 Biochemical analysis and sandwich enzyme-linked immune immunosorbent sandwich assay (ELISA)

Serum was isolated and used for biochemical analysis and sandwich enzyme-linked immunosorbent assay (ELISA). Biochemical analysis was conducted using an automatic biochemical analyzer (HITACHI, 3,100, Tokyo, Japan) with biochemical reagent kits (KOFA Biotechnology Co., Ltd., Guangzhou, China) for albumin (ALB, 220,713), calcium (Ca), creatinine (Cr, 221216), lactate dehydrogenase (LDH, 220712), low-density lipoprotein (LDL-C, 220630), high-density lipoprotein (HDL-C, 220630), sodium (Na), phosphorus P), total bilirubin (TBIL, 220630), total cholesterol (TCHO, 220630), and urea (220720). Bone sialoprotein (BSP, AD230610), Klotho, fibroblast growth factor-23 (FGF-23, AD230611), lipopolysaccharide (LPS, AD230613), and sclerostin (SOST, AD230609) levels were determined using ELISA kits (Andy Gene Biotechnology Co., Ltd., Beijing, China) according to the manufacturer’s instruction. Renal injury index can be indicated by serum amount of creatinine (Cr) and urea, and sclerosis index was calculated as follows: sclerosis index = (TCHO-[ HDL-C])/HDL-C.

### 2.4 Hematoxylin-eosin staining and von kossa staining

The harvested kidneys and thoracic aortas were fixed in 4% paraformaldehyde and sectioned. Thereafter, they were subjected to von Kossa staining for calcification analysis, following by hematoxylin-eosin staining (HE) for morphological analysis. The mean density of calcification was calculated as the ratio of integrated optical density (IOD) to the total number of pixels in the tissue region (IOD/10^6^ pixel) by Image Pro Plus 6.0 software (Media Cybernetics, Silver Spring, United States).

### 2.5 DNA extraction and 16S ribosome RNA V3-V4 region sequencing

16S rRNA sequencing targeting the V3-V4 region was performed by Personal Biotechnology Co., Ltd (Shanghai, China) following the protocol outlined by Su et al. (2018), and the bioinformatics analysis was performed on the their online platform Genescloud (https://www.genescloud). Total genomic DNAs from feces and blood samples (five in each group) were extracted using the OMEGA Soil DNA Kit (Omega Bio-Tek, Norcross, GA, USA) in a biological safety cabinet with sterile consumables. The V3-V4 region was amplified by polymerase chain reaction PCR with Q5^®^ High-Fidelity DNA Polymerase (NEB, Ipswich, MA, United States) with the 16s rRNA forward primer (5′-ACT​CCT​ACG​GGA​GGC​AGC​A-3′) and reward primer (5′-GGACTACHVGGGTWTCTAAT-3′), which incorporated sample-specific 7-bp barcodes for multiplex sequencing. The amplicon products were purified with Vazyme VAHTSTM DNA Clean Beads (Vazyme, Nanjing, China) and quantified by Quant-iT PicoGreen dsDNA Assay Kit (Invitrogen, Carlsbad, CA, United States). After pooling in equal amounts, the amplicons were subjected to pair-end 2 × 250 bp sequencing on the Illumina MiSeq platform with MiSeq Reagent Kit v3.

Quantitative Insights Into Microbial Ecology (QIIME2 2019.4) (Bolyen et al., 2019) were employed for microbiome bioinformatics. Following quality assessment, high-quality sequencing data was trimmed, assembled, and aligned against the SILVA Release 132 Database (Kõljalg et al., 2013). A phylogeny tree was constructed with FasttTree2 based on the non-singleton amplicon operational taxonomic units (OTUs) aligned with MAFFT (Katoh et al., 2002). Alpha diversity was assessed using Chao1, Shannon, Simpson, Observed species, Pelou’s evenness, and faith_pd. Beta diversity was evaluated through Bray Curtis-based principal coordinate analysis (PCoA), permutational multivariate analysis of variance (PERMANOVA), and analysis of similarities (ANOSIM). Differential taxa microbes for each group were identified through taxon-based analysis and linear discriminant analysis with effect size (LEfSe).

### 2.6 Transcriptome sequencing

Transcriptome sequencing for kidneys was conducted by Personal Biotechnology Co., Ltd (Shanghai, China), and the bioinformatics analysis was performed on the their online platform Genescloud (https://www.genescloud). Briefly, total RNA was extracted from kidneys (three samples in each group) using Trizol Reagent (Invitrogen Life Technologies, United States). After quantification and integrity testing, 3 μg of total RNA was used for sequencing sample preparation. Following purification with poly-T oligo-attached magnetic beads, the total RNAs were fragmented using divalent cations at an elevated temperature in Illumina proprietary fragmentation buffer. First-strand cDNA was synthesized using random oligonucleotides and SuperScript II, followed by second-strand cDNA synthesis using DNA Polymerase I and RNase H. Remaining overhangs were converted into blunt ends via exonuclease/polymerase activities, and the enzymes were then removed. After adenylation of the 3′ends of the DNA fragments, Illumina PE adapter oligonucleotides were ligated to prepare for hybridization. The resulting 400–500 bp cDNA fragments were purified using the AMPure XP system (Beckman Coulter, Beverly, CA, United States). Fragments with ligated adaptor molecules on both ends were enriched using Illumina PCR Primer Cocktail in a 15-cycle PCR assay. Products were purified, quantified, and, finally, sequenced on the Illumina NovaSeq 6,000 platform.

### 2.7 Real-time quantitative reverse transcription–polymerase chain reaction (RT-qPCR) analysis

Total RNA was isolated from kidney tissues using the Animal Total RNA Isolation Kit (Foregene Co., Ltd., Chengdu, China) and then reverse transcribed with SweScript All-in-One RT SuperMix (Wuhan Servicebio Technology Co., Ltd., Wuhan, China). The resulting cDNAs underwent quantitative reverse transcription–PCR (qRT-PCR) assays using universal Blue SYBR Green qRT-PCR Master Mix (Wuhan Servicebio Technology Co., Ltd., Wuhan, China) and were analyzed on a CFX Connect™ Real-Time system (Bio-Rad, NY, United States). The primer sequences for qRT-PCR are provided in Supplementary Table S1. Data were normalized with GAPDH as the internal control and analyzed using the 2^−△△Ct^ method.

### 2.8 Statistical analysis

Statistical calculations were performed in GraphPad Prism (version 9.0). Data were presented as mean ± standard deviation (SD). Testing for normal distribution was conducted before the subsequent analysis. If the data were found to fit a normal distribution, then datasets were subjected to one-way analysis (ANOVA) of variance or Student’s t-test. If the data had a non-normal distribution, then they were compared using the Mann-Whitney U test or Kruskal–Wallis test. Spearman or Pearson correlation analysis was employed to identify associations between variables.

## 3 Results

### 3.1 Calcification was observed in the kidney and thoracic aorta in the adenine diet-induced CKD rat model

**FIGURE 1:**
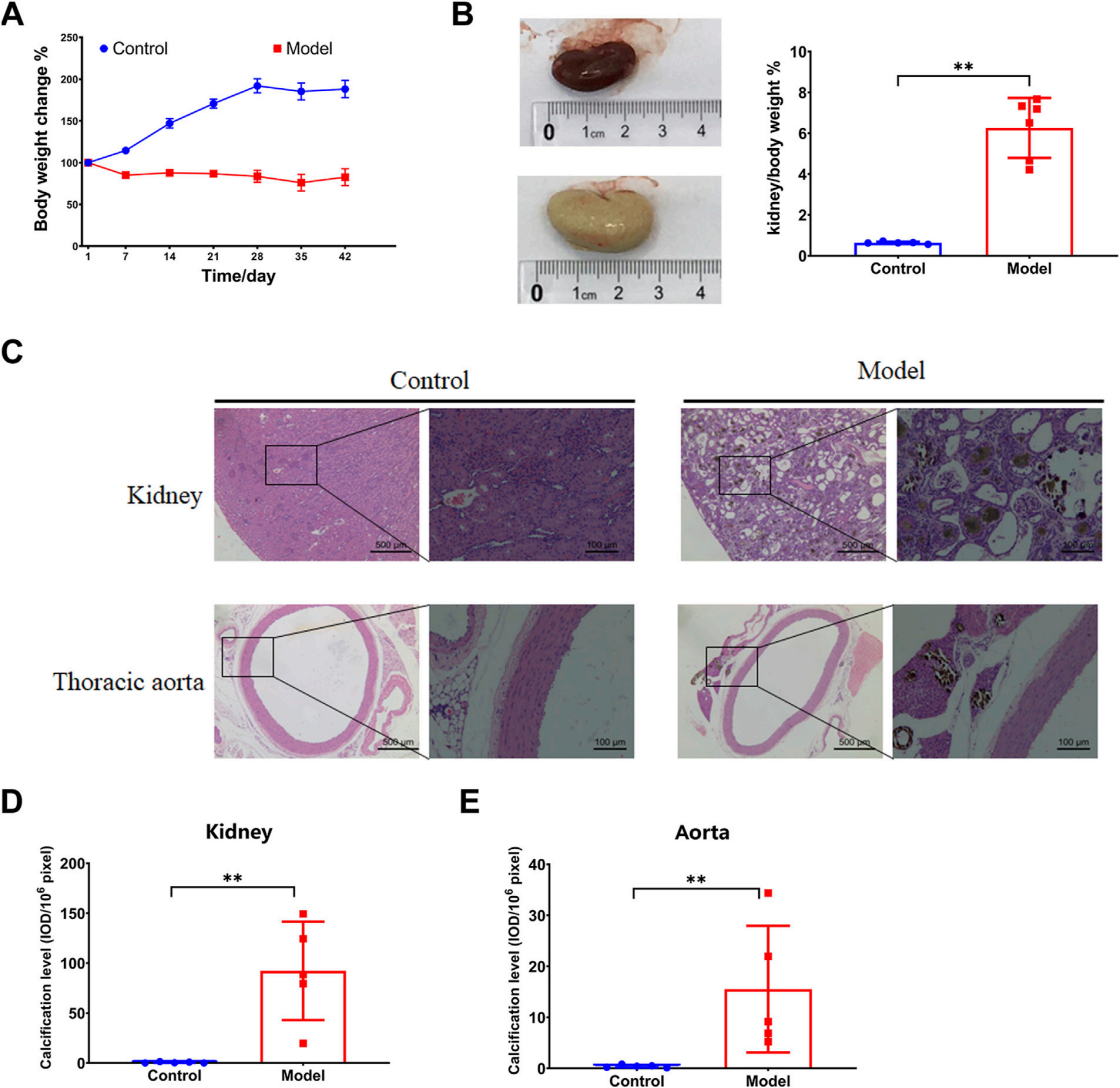
Vascular calcification in an adenine diet-induced chronic kidney disease rat model. **(A)** Body weight change. **(B)** Kidney weight index. **(C–E)** HE and calcification analysis by von Kossa staining in for kidney and thoracic aorta. N = 5. *, *p <* 0.05; **, *p <* 0.01.

After the 6-week 0.75% adenine diet, the model group exhibited hampered body weight gain (Figure 1A). The kidneys displayed noticeable swelling and whitening, accompanied by a sharp increase in kidney-to-body weight ratio (Figure 1B). Furthermore, pathological microscopy and calcification detection revealed disordered glomerular structure, dilated renal tubules, infiltration of inflammatory cells, renal interstitial fibrosis, and deposition of brownish crystals in the renal tubules induced by the 0.75% adenine diet. Apparent calcification was observed in both the kidney and thoracic aorta (Figure 1C), as corroborated by the statistical analysis (Figures 1D, E). All these findings suggest that the high adenine diet-induced chronic kidney injury leads to calcification in both the thoracic aorta and kidney, thereby exacerbating CKD.

### 3.2 Dysbiosis of gut and blood microbiota existed in CKD progression

**FIGURE 2:**
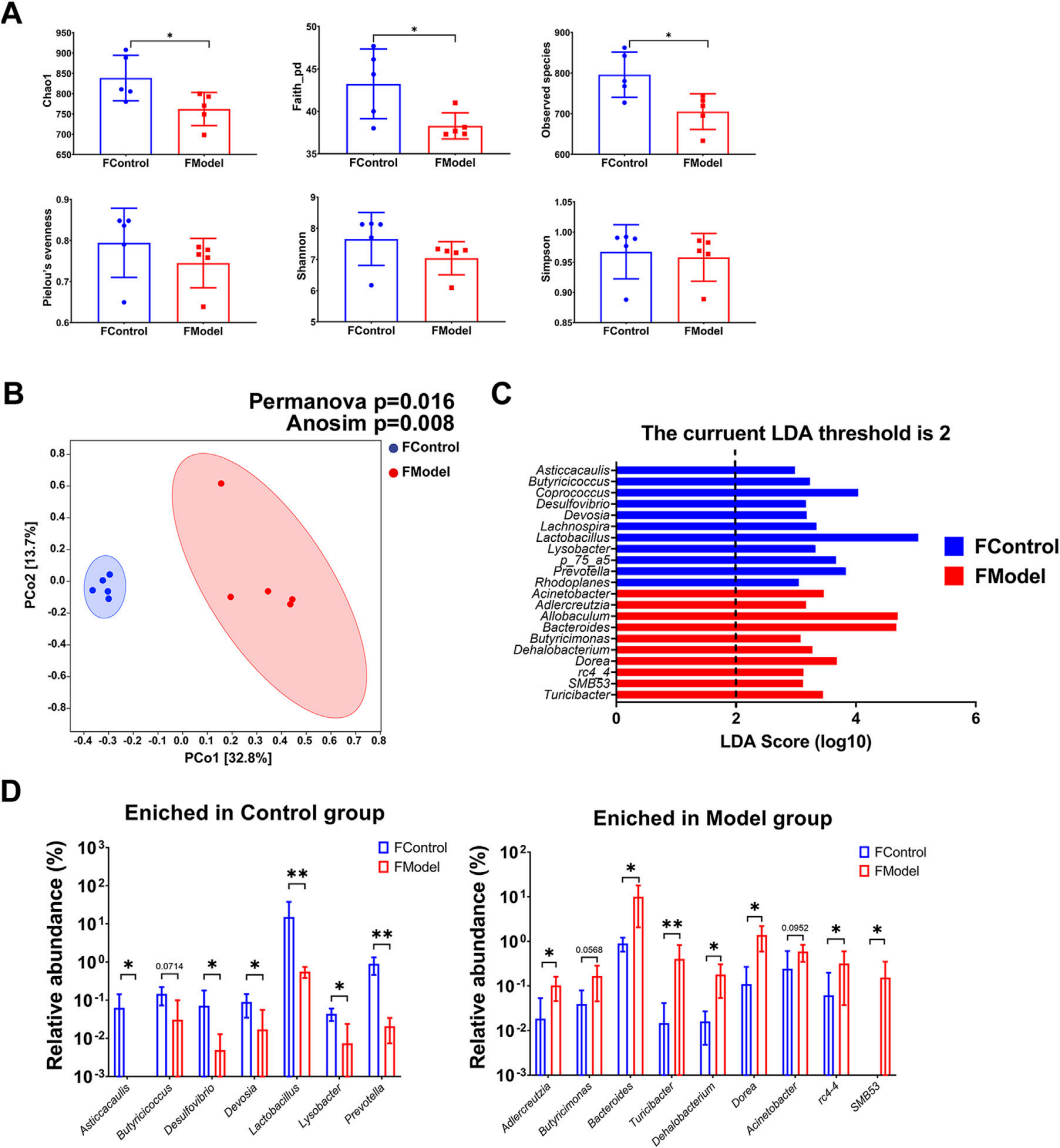
Gut microbiota characteristic of CKD rats. **(A)** Alpha diversity indices **(B)** Beta diversity presented by PCoA, and statistic analyzed by PERMANOVA and ANOSIM **(C)** LEfSe analysis **(D)** Enriched genera in Control group and Model group. N = 5. *, *p <* 0.05; **, *p <* 0.01.

Symbiotic microbiota was closely associated with the progression of CKD. Diversity of species, including alpha and beta diversities, is the critical parameter in microbiota analysis. Alpha diversity indicated the richness, diversity, and evenness within the community. Beta diversity, also named between-habitat diversity, referred tothe diversity in species composition between different habitat communities. We found a significant decrease in alpha diversity indices, including Chao1, observed species, and faith_pd, in the CKD rats. This indicates a notable disruption in evenness and species diversity in the CKD rats (Figure 2A). The beta diversity analysis showed that the Model group was markedly distinct from the Control group, as demonstrated by the PCoA. This distinction was further confirmed by PERMANOVA (*p* = 0.016) and ANOSIM (*p* = 0.008) (Figure 2B). LEfSe analysis revealed that 11 genera were enriched in the Control group, while the Model group had 10 genera (Figure 2C, Supplementary Data Sheet S1). Specifically, the relative abundance of the genera *Asticcacaulis*, *Butyricicoccus*, *Desulfovibrio*, *Devosia*, *Lactobacillus*, *Lysobacter*, and *Prevotella* was much lower in the Model group than in the Control group, and the genera *Asticcacaulis*, *Butyricicoccus*, *Desulfovibrio*, *Devosia*, *Lactobacillus*, *Lysobacter*, and *Prevotella* could barely be detected in the Model group (Figure 2D, Supplementary Data Sheet S1). On the contrary, the genera *Adlercreutzia*, *Butyricimonas*, *Bacteroides*, *Turicibacter*, *Dehalobacterium*, *Dorea*, *rc4-4*, and *SMB53* were significantly higher in the Model group. Notably, *rc4-4* and *SMB53* were almost exclusively found in the Model group (Figure 2D).

**FIGURE 3:**
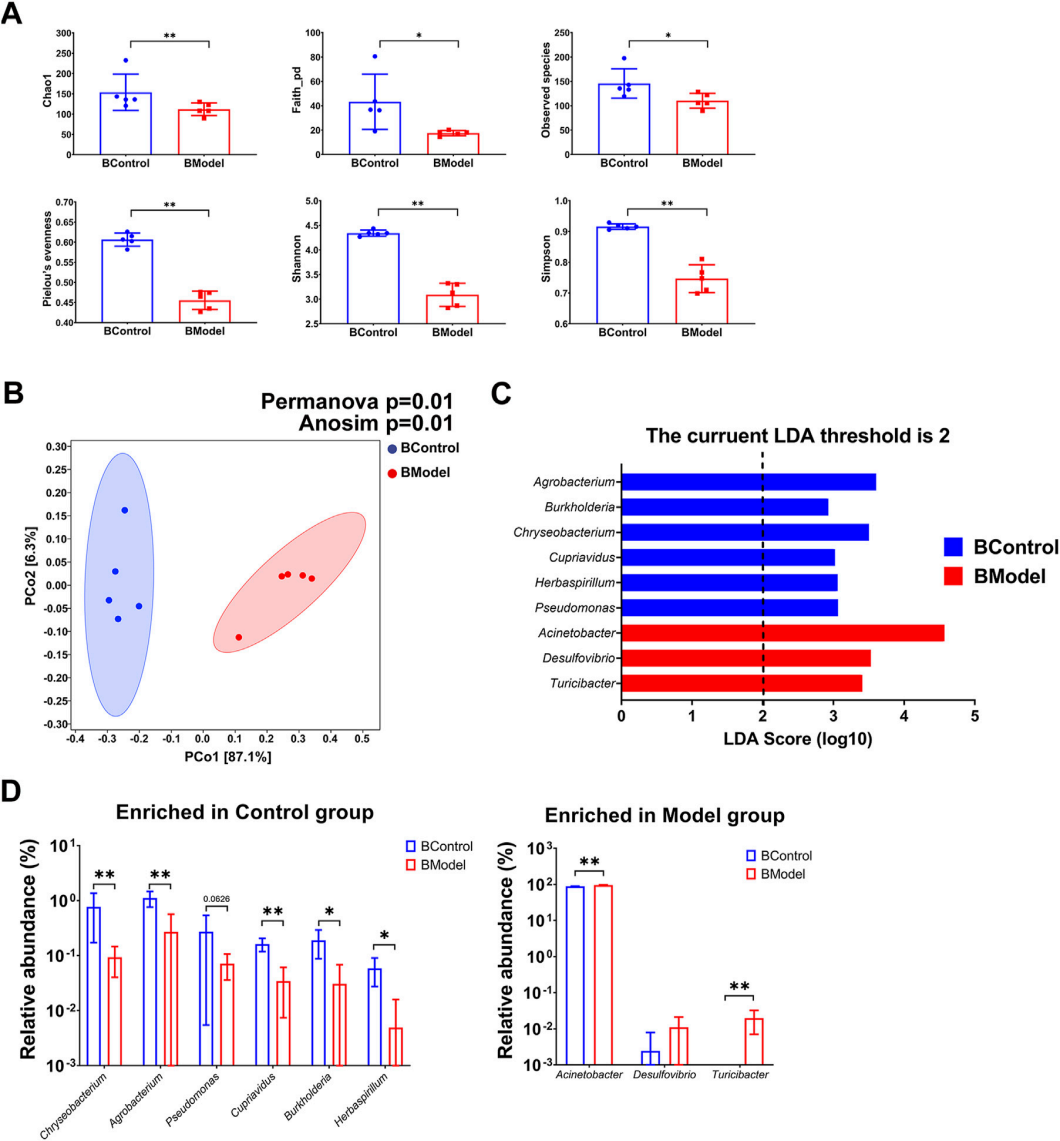
Blood microbiota characteristic of CKD rats. **(A)** Alpha diversity indices **(B)** Beta diversity presented by PCoA, and statistic analyzed by PERMANOVA and ANOSIM **(C)** LEfSe analysis **(D)** Enriched genera in Control group and Model group. N = 5. *, *p <* 0.05; **, *p <* 0.01.

Interestingly, it was observed that the blood microbiota also displayed dysbiosis in the progression of CKD. Alpha diversity of the blood microbiota in the Model group was significantly decreased, indicated by lower Chao1, Shannon, Simpson, Observed species, Pelou’s evenness, and faith_pd indices (Figure 3A). Beta diversity analysis, as shown by PCoA along with PERMANOVA (*p* = 0.01) and ANOSIM (*p* = 0.01), indicated a notable difference between the Control and Model groups (Figure 3B). LEfSe analysis revealed that six genera were enriched in the Control group, while only three genera were higher in the Model group (Figure 3C, Supplementary Data Sheet S1). Among the differential genera, the relative abundance of *Chryseobacterium*, *Agrobacterium*, *Cupriavidus*, *Burkholderia*, and *Herbaspirillum* was lower in the Model group, while that of *Acinetobacter* and *Turicibacter* significantly increased (Figure 3D). Noteworthy, *Acinetobacter* constituted a substantial portion of the blood microbiota in both groups (87.86% ± 1.69% in the Control group vs. 96.02% ± 1.27% in the Model group, *p* < 0.01) and exhibited an increasing trend in the gut microbiota of CKD rats (0.24% ± 0.36% in the Control group vs. 0.59% ± 0.24% in the Model group, *p* = 0.0952). Additionally, *Turicibacter* was exclusively detected in the Model group. All these findings suggest that dysbiosis of the symbiotic microbiota, both in the gut and blood, is a promising characteristic of CKD.

### 3.3 Dysbiosis of gut and blood microbiota was closely related to the disorder of biochemical indices and calcification-related factors

**FIGURE 4:**
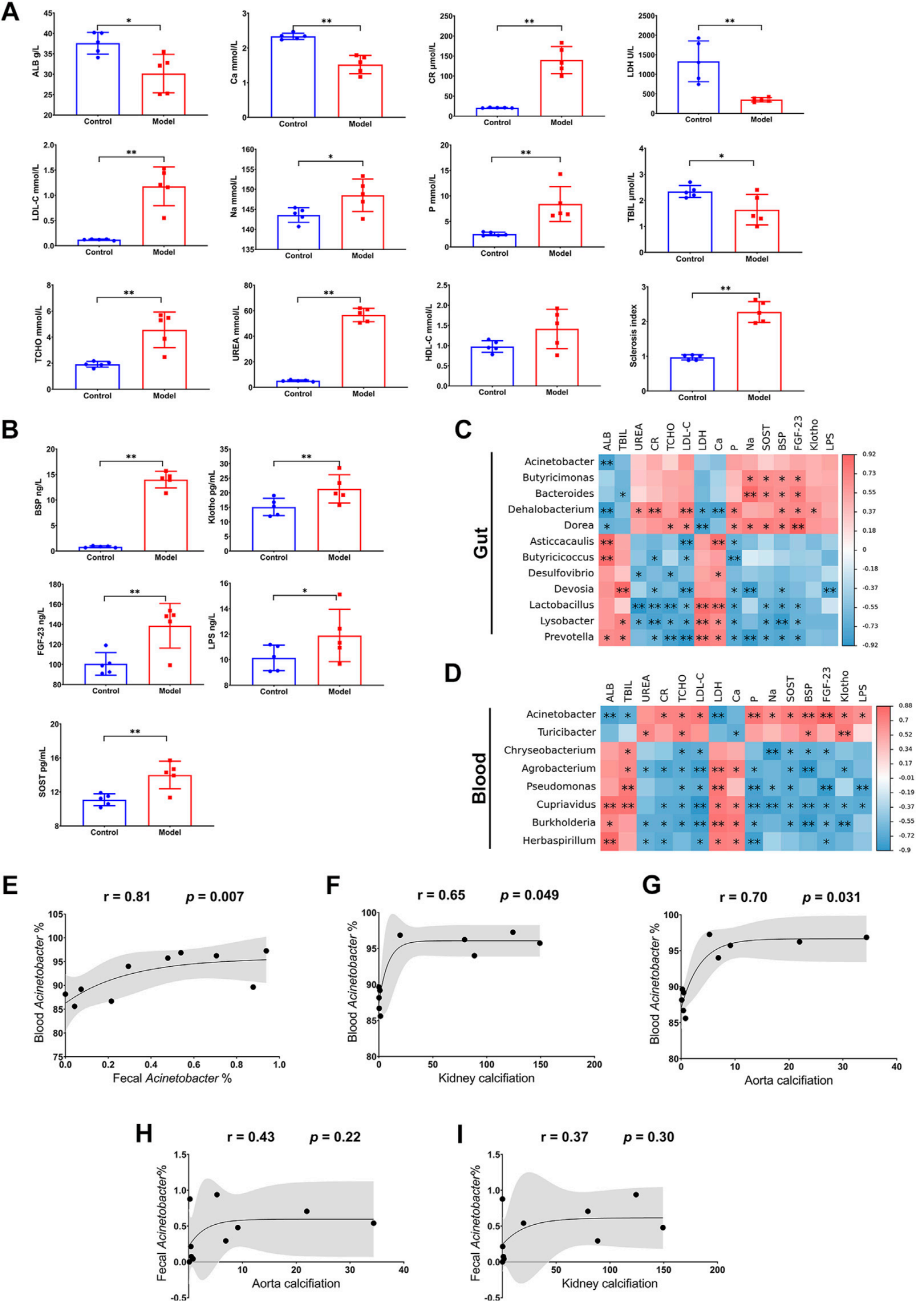
Relationship between microbiota dysbiosis and disorder of biochemical indices and calcification-related factors. **(A)** Biochemical analysis for ALB, Ca, CR, LDH, LDL-C, Na, P, TBIL, TCHO, UREA. **(B)** ELISA for BSP, Klotho, FGF-23, LPS, and SOST. **(C,D)** Correlation analysis for biochemical indices and calcification-related factors and differential microorganisms in gut and blood. **(E)** Correlation analysis for *Acinetobacter* genus in gut and blood. **(F,G)** Correlation analysis for blood *Acinetobacter* genus and calcification in kidney and thoracic aorta. **(H,I)** Correlation analysis for fecal *Acinetobacter* genus and calcification in kidney and thoracic aorta. N = 5. *, *p <* 0.05; **, *p <* 0.01.

Through serum chemical analysis, it was observed that ALB, Ca, LDH, and TBIL were remarkably decreased after the high adenine diet, while Cr, LDL-C, Na, P, TCHO, Urea, as well as sclerosis index, were significantly increased (Figure 4A). Furthermore, several calcification-related factors in the serum, including BSP, Klotho, FGF-23, and SOST, showed significant surges in the Model group (Figure 4B). Moreover, LPS, an endotoxin from microbes, was also increased in the Model group (Figure 4B). These results suggest that the severe kidney injury in CKD is accompanied by notable dysregulation of biochemical indices and calcification-related factors.

Remarkably, the disorder of biochemical indices and calcification-related factors was significantly correlated with the enriched microorganisms in the Model group in both the gut and blood. Regarding the gut microbiota, *Dehalobacterium* and *Dorea* were negatively correlated with ALB and LDH, but positively correlated with most of the increased biochemical indices (Urea, Cr, TCHO, and LDL-C) and calcification-related factors (P, Na, SOST, BSP, FGF-23, and Klotho); *Bacteroides* was negatively correlated with TBIL, but positively correlated with Na, SOST, BSP, FGF-23, and Klotho; finally, *Butyricicoccus* showed a positive correlation with Na, SOST, BSP, and FGF-23 (Figure 4C). As for the blood microbiota, the enriched microorganisms displayed even more correlation with the disorder of biochemical indices and calcification-related factors. *Acinetobacter* was negatively correlated with ALB, TBIL, and LDH, but positively correlated with most of the other biochemical indices (Cr, TCHO, LDL-C, P, and Na), all the calcification-related factors (SOST, BSP, FGF-23, and Klotho), and LPS. By contrast, *Chryseobacterium*, *Agrobacterium*, *Pseudomonas*, *Cupriavidus*, *Burkholderia*, and *Herbaspirillum* were positively correlated with ALB, TBIL, LDH, or Ca, but negatively correlated with the other indices and factors (Figure 4D). Intriguingly, *Acinetobacter* in blood exhibited more evident correlations with indices and factors than that in the gut, although both of them were evidently negatively associated with ALB (Figures 4C, D). Particularly, *Acinetobacter* in blood was not only positively correlated with that in feces (r = 0.81, *p* = 0.007, Figure 4E) but also had a positive association with calcifications in the kidney (r = 0.65, *p* = 0.049, Figure 4F) and thoracic aorta (r = 0.70, *p* = 0.031, Figure 4G). Nevertheless, change of *Acinetobacter* in feces was not closely related to the calcification in kidney or that in aorta (Figures 4H, I). These results indicated that CKD not only results in a disorder of biochemical indices and calcification-related factors, but also comes with dysbiosis of the symbiotic microbiota, especially the disruption of blood microbiota homeostasis. Notably, the enrichment of *Acinetobacter* in blood could potentially serve as a risk factor for CKD and the accompanying VC.

### 3.4 Dysbiosis of gut and blood microbiota was associated with the affected mineral absorption function in CKD

**FIGURE 5:**
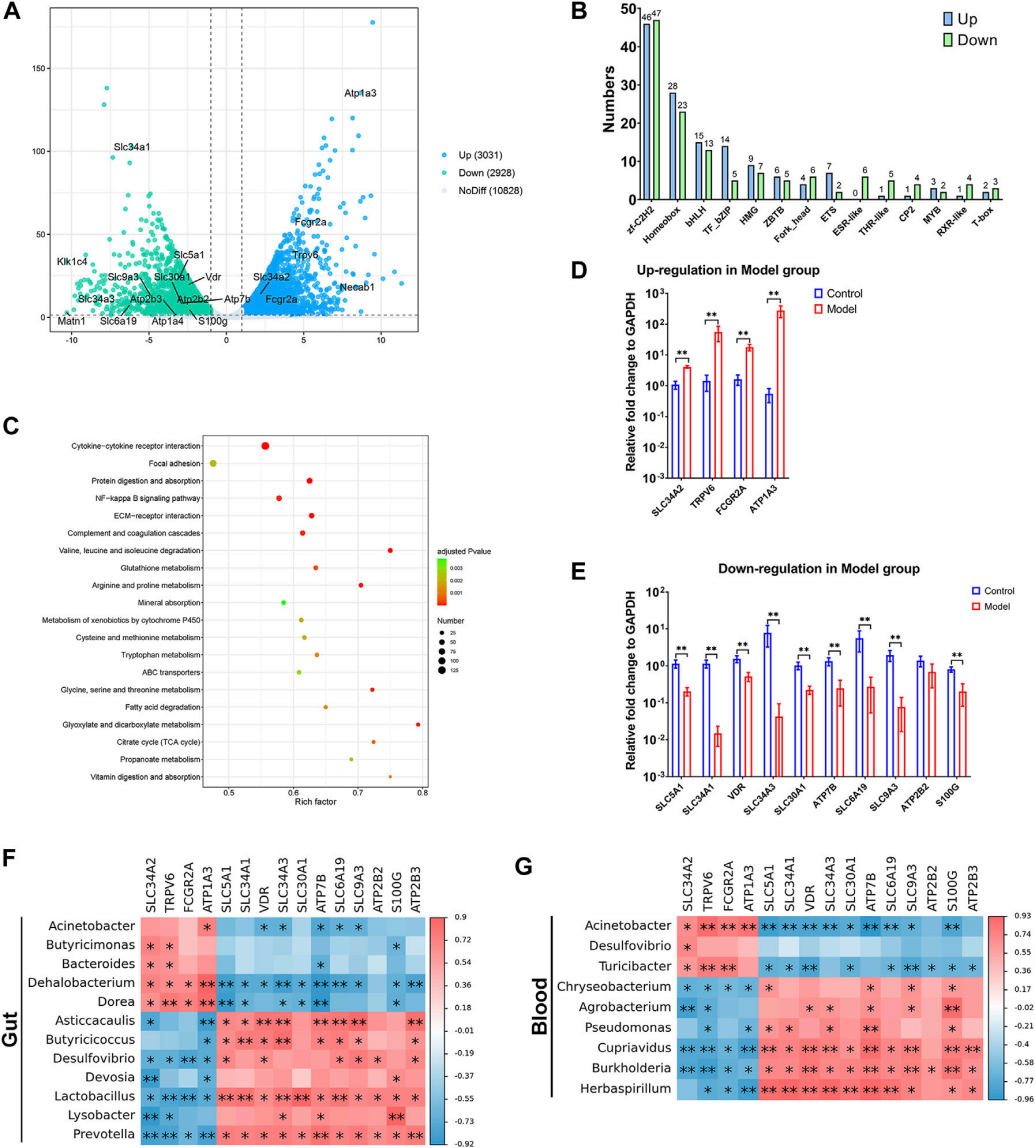
Transcriptome of kidney was intensively affected in the progression of CKD. **(A)** differentially expressed genes (DEGs) showed by volcano plot. **(B)** DEGs that belongs to transcription factor families. **(C)** Signaling pathway enrichment for DEGs based on KEGG. **(D,E)** Up- and downregulated DEGs in mineral absorption signal pathway in Model group. **(F,G)** Correlation analysis for DEGs in mineral absorption signal pathway and differential microorganisms in gut and blood. N = 5, *, *p <* 0.05; **, *p <* 0.01.

We conducted transcriptome sequencing on kidney tissue to investigate the underlying mechanisms in the progression of CKD. As depicted in Figure 5A, there were 3,031 upregulated genes and 2,928 downregulated genes in the kidneys of the Model group compared to the Control group (Figure 5A, Supplementary Data Sheet S2). Some of the differentially expressed genes (DEGs) belong to various transcription factor families. For instance, there were 46 upregulated DEGs and 47 downregulated DEGs in the zf-C2H2s family, and 28 upregulated DEGs and 23 downregulated DEGs in the Homeobox family (Figure 5B, Supplementary Data Sheet S2). Through pathway enrichment analysis in the Kyoto Encyclopedia of Genes and Genomes (KEGG), the DEGs were implicated in 46 signaling pathways (adjusted *p* < 0.05, Supplementary Data Sheet S2). Among the top 20 pathways, it is particularly noteworthy that mineral absorption was significantly affected (Figure 5C), given that dysregulation of mineral homeostasis is a hallmark of CKD. In summary, four genes (SLC34A2, TRPV6, FCGR2A, and ATP1A3) were upregulated under high adenine diet-induced CKD (Figure 5D), while 10 genes (SLC5A1, SLC34A1, VDR, SLC34A3, SLC30A1, ATP7B, SLC6A19, SLC9A3, ATP2B2, and S100G) were downregulated (Figure 5E).

Interestingly, most of the DEGs exhibited positive associations with the enriched gut microorganisms, while they were negatively associated with the depleted ones. For example, all the upregulated DEGs in the Model group were accompanied by a higher relative abundance of *Dehalobacterium* and *Dorea*, while having a lower abundance of *Desulfovibrio*, *Lactobacillus*, and *Prevotella*. Most of the downregulated DEGs were accompanied by enriched *Dehalobacterium* and *Dorea*, whereas being depleted in *Asticcacaulis*, *Butyricicoccus*, *Lactobacillus*, and *Prevotella* (Figure 5F).

A similar correlation between DEGs and microbiota was also found in the blood. The enriched *Acinetobacter* and *Turicibacter* in the Model group were positively related to most of the upregulated DEGs and negatively related to the downregulated DEGs, while the depleted *Chryseobacterium*, *Cupriavidus*, *Burkholderia*, and *Herbaspirillum* showed a contrasting relationship with the aforementioned DEGs (Figure 5G). Notably, the DEGs exhibited a stronger association with *Acinetobacter* in the blood than in the gut, indicating that the increased presence of *Acinetobacter* in the blood could potentially be a risk factor in the progression of CKD.

## 4 Discussion

There is now a consensus that the commensal microbiome plays a vital role in the progression of CKD. Recent studies have demonstrated that uremic toxins derived from microbes, such as P-cresyl sulfate (PCS) and trimethylamine N-oxide (TMAO), have been implicated as risks in CKD and subsequent cardiovascular events (Meijers et al., 2010; Missailidis et al., 2016). Increases in PCS and indoxyl sulfate (IS) have been linked to the decline of renal function (Barrios et al., 2015). Study by Ren et al. (2020) found that microbial diversity is notably reduced in CKD patients. The community is characterized by an enrichment of genera *Klebsiella* and Enterobacteriaceae, while *Blautia* and *Roseburia* are reduced. They also proposed an optimal five-microbial panel for identifying CKD. Similarly, our results showed a reduced gut microbiota diversity in adenine diet-induced CKD rats. Beta diversity was distinguished by a higher relative abundance of *Adlercreutzia*, *Butyricimonas*, *Bacteroides*, *Turicibacter*, *Dehalobacterium*, *Dorea*, *rc4-4*, and *SMB5*. The distinctive genera found in CKD rats differed from those in CKD patients enrolled in the study of Ren et al. (2020), possibly due to the varied causes and stages of CKD in patients as compared to the adenine diet-induced CKD rats.

Surprisingly, our data revealed that the difference in blood microbiota between the Control group and the Model group was greater than that in gut microbiota. In summary, all of the alpha diversity indices (Chao1, Shannon, Simpson, Observed species, Pelou’s evenness, and faith_pd) were significantly decreased, and the microbial community (beta diversity) of the Model group was distinct from that of the Control group with a notable statistically significant difference. Notably, among the differential genera, *Acinetobacter* comprised a large portion of the blood microbiota in both groups. It not only showed a significant enrichment in blood microbiota but also tended to increase in the gut. Such microbiome dysbiosis was accompanied by significantly higher LPS levels. While no specific study has explored the blood microbiota of CKD patients, one study reported that *Acinetobacter* is increased in the blood microbiota of patients with inflammatory bowel disease (Xu et al., 2023) and multiple system atrophy with predominantly cerebellar ataxia (Du et al., 2019). In accordant with the findings by Shah et al. (2019) and Merino-Ribas et al. (2022), results from the present study found the microbiota dysbiosis (both gut and blood) in the progression of CKD, especially the enrichment of *Acinetobacter* genus in the blood. However, the exact causality between microbiota dysbiosis and CKD required further exploration.

VC is one of the most critical complications in CKD progression, wreaking havoc on various physiological functions, notably those related to the cardiovascular system. VC involves the improper biomineralization occurring within the soft tissues of the vascular system. Interestingly, in CKD, demineralization in bone and mineralization in the vasculature happen simultaneously, a phenomenon often referred to as the “calcification paradox” or “bone-vascular axis” (Evenepoel et al., 2019). Damage to vascular smooth muscle, along with processes such as apoptosis and transdifferentiation into cells akin to osteoblasts, triggers the expression of bone-related proteins and leads to the mineralization of the extracellular matrix, ultimately resulting in VC (Li et al., 2022). In CKD patients, disturbances in calcium and phosphorus metabolism often lead to an increase in parathyroid hormone and FGF-23, along with a decrease in its co-receptor Klotho. This accelerates bone transformation and ultimately exacerbates VC (Pazianas and Miller, 2021). BSP is an extracellular matrix protein that is highly sulfated, phosphorylated, and glycosylated. It is produced and secreted by osteoblasts, osteoclasts, and other bone-related cells. Elevated levels of BSP are often used as a marker to assess the degree of differentiation and osteogenic activity in osteoblasts (Ogata, 2008) and VC (Furmanik et al., 2021). SOST is an inhibitor of bone formation derived from osteocytes and is increased in cases of kidney failure, particularly in CKD patients with VC (Caus et al., 2023). Consequently, factors such as FGF-23, SOST, and Klotho are believed to be emerging players in the CKD-induced VC and its association with cardiovascular mortality (Kaur and Singh, 2022). In our present study, we observed not only dysregulation in various biochemical indices (such as decreased ALB, LDH, and TBIL, and increased Cr, LDL-C, Na, TCHO, and Urea), but also an imbalance in calcium-phosphorus metabolism in the CKD-afflicted rats (lower levels of Ca and higher levels of P). This was concurrent with significant surges in BSP, FGF-23, and SOST levels. Furthermore, verification through transcriptome sequencing in kidney tissue using qRT-PCR revealed a substantial impact on mineral absorption function, as evidenced by four upregulated genes and ten downregulated genes. All these findings suggest that CKD induced by the adenine diet disrupts mineral ion metabolism, particularly calcium-phosphorus metabolism. This disruption, in turn, triggers dysregulation in several controlling factors, such as BSP, FGF-23, and SOST, thereby contributing to VC.

The increase of *Acinetobacter* in the blood is also a potential risk factor for VC in the progression of CKD. *Acinetobacter* is an aerobic Gram-negative bacterium known for its ability to accumulate P and precipitate Ca. Firstly, *Acinetobacter* can regulate P concentration based on oxygen levels in the environment. It accumulates P under aerobic conditions and releases it under anaerobic conditions (Tandoi et al., 1998). Secondly, *Acinetobacter* has been found to induce Ca^2+^ influx and disrupt cellular Ca^2+^ homeostasis, ultimately leading to cell death in the lung tissue (Smani et al., 2011). Data indicate that, in addition to its positive correlation with gut levels, *Acinetobacter* in the blood is also positively associated with calcifications in the kidney and thoracic aorta. The enrichment of *Acinetobacter* is accompanied by increases in calcification factors (BSP, FGF-23, and SOST), LPS, and P. Patients with CKD often experience body fluid retention and urea accumulation. The colon takes over from the kidneys as the main site for uric acid excretion, the end product of urea. Metabolites of uric acid not only alter the growth environment of gut microbes (Hobby et al., 2019), but also compromise the epithelial barrier, increasing mucosal permeability by inducing the contraction and endocytosis of transcellular tight junction proteins (Chen et al., 2019). As a result, the local inflammation was initiated, stimulating the influx of white blood cells and triggering cytokine production (Vaziri et al., 2013). Some microorganisms are translocated into the bloodstream along with endotoxin and fragments, ultimately increasing the risk of sepsis during CKD (Hobby et al., 2019). As demonstrated by our data, it is likely that *Acinetobacter* may translocate into the bloodstream during CKD due to elevated environmental pH and P concentration. Consequently, the enriched *Acinetobacter* promotes Ca-precipitation in vascular smooth muscle cells, thereby exacerbating VC.

A limitation in our study is the exact causality between microbiota dysbiosis and CKD, as well as the accompanying VC, remains unclear. And mechanism underlying the role of *Acinetobacter* in blood in the progression of CKD also required further exploration. Moreover, the increase in *Klotho* in CKD rats contradicts its protective role reported in several studies (Caus et al., 2023; Martín-Vírgala et al., 2023). Further investigation is needed to determine whether this paradox arises from the differences between nephrectomy-induced CKD and adenine diet-induced CKD. Overall, analysis on the patients with CKD is needed in the future study on the relationship between microbiota dysbiosis and CKD.

## 5 Conclusion

In summary, our study underscores that in the progression of adenine diet-induced CKD, chronic kidney injury is accompanied with calcification in the thoracic aorta and kidney. The concurrent dysbiosis of symbiotic microbiota, including both gut and blood microbiota, emerges as a promising characteristic of CKD. Notably, the enrichment of *Acinetobacter* in the blood may serve as a potential risk factor for CKD and the accompanying VC. This may be attributed to the rise in environmental pH and P concentration during CKD, enabling *Acinetobacter* to facilitate Ca-precipitation in vascular smooth muscle cells, thereby aggravating VC.

## Data Availability

All data generated during the current study are included in this article and its supplementary information files, and they are available from the corresponding authors upon reasonable request. The sequences generated and analyzed in this study were uploaded to the NCBI Sequence Read Archive (SRA) data repository with project numbers PRJNA1022598 (16S rRNA sequencing raw data) and PRJNA1023034 (Transcriptome sequencing raw data).
