# Supplementary material for: Role of symbiotic microbiota dysbiosis in the progression of chronic kidney disease accompanied with vascular calcification
Source: Front Pharmacol. 2024 Jan 5;14:1306125. doi: 10.3389/fphar.2023.1306125 (PMC10796560; doi:10.3389/fphar.2023.1306125)
Supplement: Supplementary file 5 [file Table6.DOCX]

Table S 3 Primers for real-time PCR

| Gene name | Primer sequence | | Product length |
| --- | --- | --- | --- |
| Slc34a1 | Sense | GACAGAAATGAGTTCAGAAGGGCA | 125 |
|  | Antisense | AGGTTGGTCAGTTTCTCCAGGTA |  |
| Slc34a1 | Sense | ACCGCCCTTTACACCATCACA | 106 |
|  | Antisense | AAGCAAACCCAGTCAGGATAAAA |  |
| Slc34a1 | Sense | CCGTTGATGCTAGGCTTCCT | 101 |
|  | Antisense | TTGAAGATGTCCCCAGCCAC |  |
| VDR | Sense | GATGACCCGTCTGTGACTCTGG | 203 |
|  | Antisense | GTGAAAGACTGGTTGGAGCGT |  |
| Slc34a3 | Sense | CAAGATGGCCGGAGACATT | 180 |
|  | Antisense | CCCATGATGATAGGGACAGACG |  |
| Slc30a1 | Sense | TACTTCTTTACACGACGTACCCAC | 129 |
|  | Antisense | GGACTTCCTCCACTCCTTCAACA |  |
| ATP7B | Sense | CCATGCCTGTCACCAAGAAA | 195 |
|  | Antisense | GGACGAAATATCCACTGAACCG |  |
| GAPDH | Sense | CTGGAGAAACCTGCCAAGTATG | 138 |
|  | Antisense | GGTGGAAGAATGGGAGTTGCT |  |
| TRPV6 | Sense | CACTGCTCATGCTCAACCTCCTA | 332 |
|  | Antisense | ACCAAAGGGTCGTGCCATCT |  |
| Slc9a3 | Sense | GGCTTCGTCTTTGTCATTTCCTA | 124 |
|  | Antisense | CTGAGATGTTGGCCTTCACGTAC |  |
| ATP2B2 | Sense | TTTCGGGCAAAACTTCATACC | 163 |
|  | Antisense | GGCACATCCTTCATTGCTCTCT |  |
| Slc6a19 | Sense | ACAAGGCCCAGTACATGCTCA | 184 |
|  | Antisense | TTGCGTAGCCTCTGTCCGA |  |
| S100g | Sense | CTGGATAAGAACGGTGATGGAGA | 87 |
|  | Antisense | CTCCTTCTTCTGGCTTCATTGTG |  |
| Fcgr2a | Sense | TTGAGGTGTCACGGCTGGA | 258 |
|  | Antisense | CACTAGGCAGAAAGCGGCAT |  |
| Atp1a3 | Sense | GTCCTCGGGTTCCGTAAAGC | 169 |
|  | Antisense | ACAGCGGTCCAGAATGCGT |  |
| Atp2b3 | Sense | GAGAAAGGCGAGATAGAACAGGA | 109 |
|  | Antisense | CAATTATGCCTTTGACCAGAGTATG |  |
